# Circulating miR-25-3p and miR-451a May Be Potential Biomarkers for the Diagnosis of Papillary Thyroid Carcinoma

**DOI:** 10.1371/journal.pone.0132403

**Published:** 2015-07-13

**Authors:** Min Li, Qinbin Song, Hang Li, Yi Lou, Lili Wang

**Affiliations:** 1 Department of Endocrinology, the First Affiliated Hospital, China Medical University, Shenyang, 110001, China; 2 Department of Thyroid Surgery, the First Affiliated Hospital, China Medical University, Shenyang, 110001, China; 3 Department of Genetics, China Medical University, Shenyang, 110001, China; 4 Medical Research Center of Shengjing Hospital, China Medical University, Shenyang, 110004, China; SAINT LOUIS UNIVERSITY, UNITED STATES

## Abstract

**Objective:**

There is no effective and reliable biomarker to distinguish benign thyroid nodules from papillary thyroid carcinomas (PTC). This study aimed at examining the levels of plasma miRNAs in patients with PTC or benign nodules to explore the potential miRNA biomarkers for PTC.

**Patients and Methods:**

Genome-wide plasma miRNA expression profiles were determined by the miRNA Microarray and the significantly higher levels of miRNAs were validated in plasma and tissues by quantitative RT-PCR. The levels of two miRNAs were further tested in seven patients before and after tumor excision and the potential values for the diagnosis of PTC were evaluated by receiver operating characteristic curve (ROC).

**Results:**

In comparison with that in the patients with benign nodules, eight significantly higher and three lower levels of plasma miRNAs were detected in the PTC patients. Further validation indicated that the levels of plasma miR-25-3p, miR-451a, miR-140-3p and let-7i were significantly higher in the PTC cases than in those with benign nodules or the healthy controls. Significantly higher levels of miR-25-3p and miR-451a were detected in the thyroid tissues from the PTC patients. The levels of plasma miR-25-3p and miR-451a in seven patients significantly decreased after tumor excision. ROC analyses revealed that the levels of plasma miR-25-3p at cut-off 1.41 and miR-451a at 1.38 had sensitivity of 92.8% and 88.9%, and specificity of 68.8% and 66.7% for distinguishing PTC from benign nodules, respectively.

**Conclusion:**

Our findings suggest that the levels of plasma miR-25-3p and miR-451a may be valuable for the diagnosis of PTC.

## Introduction

Thyroid cancer is the most common endocrine malignant tumor and includes well-differentiated papillary thyroid carcinoma (PTC, 80% of thyroid cancers), follicular thyroid carcinoma (15%), poorly differentiated thyroid carcinoma (<1%) and anaplastic thyroid carcinoma (<2%) [1]. PTC is comprised of several variants, such as follicular variant, the diffuse sclerosing variant, and tall/columnar variant. PTC should be differentially distinguished from benign thyroid nodule (thyroid adenoma and classical nodular goiter) in the clinic. Currently, patients with suspicious thyroid nodules are usually examined by ultrasound, computed tomography (CT). Subsequently, patients with suspicious PTC are examined by pre-operative ultrasound-guided fine-needle aspiration cytology (FNAC) and intraoperative pathological examination of the frozen sections. However, PTC may be confused pathologically with benign papillary hyperplasia of the thyroid gland [2]. Recently, cytokeratin 19 (CK19), thyroglobulin (TG), antigen KI-67 (Ki67), Calcitonin, thyroid transcription factor 1 (TTF-1), v-raf murine sarcoma viral oncogene homolog B1 (BRAF), RET, anti-human mesothelial cell antibody (HBME-1), and galectin-3 have been suggested for distinguishing malignant from benign thyroid lesions in the clinic, which have improved the accuracy in the preoperative diagnosis of PTC [3–7]. Unfortunately, inadequate FNA sampling can lead to the failure in cytological diagnosis and may require a repeated aspiration [8]. The repeated aspiration may be dangerous for patients with PTC due to its invasive nature. A new biomarker is urgently needed to distinguish malignant from benign thyroid lesions in the clinic.

An ideal tumor marker should be measured easily, reliably, using a minimally invasive assay with high sensitivity and specificity. miRNAs are emerging as important modulators in cellular pathways, and they appear to play a key role in tumorigenesis. The miRNA in human blood is remarkably stable and is insensitive to endogenous RNase activity. The miRNAs in body fluids released from malignant cells and their intracellular miRNAs are potential biomarkers for the diagnosis of cancers [9,10]. Previous studies have shown that circulating cancer-associated miRNAs in plasma and serum samples are readily measured and they can effectively discriminate cancer patients from healthy controls [9,10]. Recent studies have analyzed miRNA expression in different types of thyroid tumor tissues and the results have demonstrated that miRNA deregulation occurs in cancer tissues [11–13]. Indeed, miR-146, miR-221, miR-222, miR-21 and miR-181 in PTC are significantly higher than that in non-tumor tissues or a benign proliferative multinodular goiter [11–13]. Furthermore, higher levels of serum let-7e, miR-151-5p, and miR-222 and up-regulated miR-151-5p and miR-222 expression are detected in the thyroid tissues of patients with PTC and they can effectively differentiate malignant and benign thyroid tumors with a high sensitivity and specificity in the Southern China [14]. Another study reveals that higher levels of circulating miR-222 and miR-146b are related to the development of PTC and their higher expression in the thyroid are associated with PTC recurrence [15]. Interestingly, the levels of circulating miR-95 and miR-190 are sensitive biomarkers for the differential diagnosis of PTC from benign thyroid tumors in Caucasians [16]. These findings indicate that varying miRNAs are sensitive biomarkers for the pre-operative diagnosis of PTC patients in different regions or ethnic backgrounds. Notably, there is a significantly ethnical difference between the Southern and Northern China. There is no information on which circulating miRNAs can serve as biomarkers for the preoperative diagnosis of PTC in Northern China.

In the present study, the levels of plasma miRNAs in patients with PTC or benign nodules were measured by a miRNA Microarray and the significantly varying levels of plasma miRNAs were validated in patients with PTC, with benign nodules or healthy controls by quantitative RT-PCR. Subsequently, the increased levels of plasma miR-25-3p and miR-451a were further characterized for their expression in the thyroid tissues from the patients with PTC or benign nodules. The levels of plasma miR-25-3p and miR-451a in seven PTC patients were examined before and after tumor excision and the values of plasma miR-25-3p and miR-451a measurements in distinguishing PTC from benign nodules were analyzed by the receiver operating characteristic curve (ROC). Our findings suggest that the levels of plasma miR-25-3p and miR-451a may be valuable for the diagnosis of PTC in Northern China.

## Subjects and Methods

### Study population and testing samples

A total of 56 patients with primary PTC, 95 patients with benign thyroid nodules, and 10 age- and gender-matched healthy controls were recruited at Department of Thyroid Surgery of the First Affiliated Hospital, China Medical University (Shenyang, China). Among 56 PTC patients (38 at TNM stage I/II, 18 at TNM stage III/IV), 44 patients had the classical variant of PTC, five with the follicular variant of PTC, three with the diffuse sclerosing variant of PTC, and four with tall/columnar cell PTC. Among 95 patients with benign thyroid nodules, 61 patients showed a thyroid adenoma and 34 patients had a classical nodular goiter. These 10 control subjects had no current or a history of thyroid disease. All patients received surgical resection of PTC or thyroid nodules and their thyroid tissue specimens were used for the pathological examination. A portion of tissue specimens from 27 PTC patients and 43 non-tumor patients were immediately frozen for preparing total RNA. In addition, blood samples were collected from all subjects before surgery and some blood samples were also collected from seven patients after tumor resection for preparing plasma samples.

### Ethics Statement

Written informed consent was obtained from individual participants and the experimental protocol was approved by the Ethics Committee of China Medical University (2013PS08K).

### Plasma miRNA profiling and data analysis

Plasma samples were randomly selected from three patients with PTC (one male, two females, age: 47.67± 1.15 years), and three with benign thyroid nodules (one male, two females, age: 45.67 ± 8.96 years) and were tested individually for the levels of miRNAs by the miRNA microarray using the Agilent Human miRNA Microarray Kit Release 19.0, which contains probes for 2006 human miRNAs from the Sanger miRBase V19.0 (Agilent, Sanra Clare, USA), according the manufacturers’ instruction. Briefly, total RNA was extracted from individual plasma samples and 100 ng total RNA of each sample was used as inputs for sample labeling and hybridization, following the manufacturers’ protocol (Agilent). The arrays were washed and scanned using a microarray scanner (G2565BA, Agilent). The intensity of each hybridization signal was evaluated by Agilent extraction software v9.5.3. The labeling, hybridization, and scanning were performed at Shanghai Biochip (Shanghai, China). The microarray image information was converted into spot intensity values using Scanner Control Software Rev. 7.0 (Agilent). The net signals were exported directly into the GeneSpring GX11.0 software (Agilent) for quantile normalization and further analysis. The differential expression of a miRNA was considered when there was a three-fold difference in the miRNA expression levels between these two groups and statistically significant difference (p < 0.05) using the DiffGene. Differentially expressed miRNAs were identified as those with false discovery rates (FDR) of <5% as per the Benjamini-Hochberg method. Hierarchical clustering of the differential expression of miRNAs between these two groups was analyzed by the Pearson Correlation analysis.

### Sample processing and total RNA isolation

Fasting blood samples were collected from all subjects and were centrifuged at 1600 g for 10 min at 10°C to prepare individual samples. The resulting plasma samples were further centrifuged at 16,000 g for 20 min at 10°C to remove cell debris and fragments. Subsequently, total RNA was extracted from 500 μl plasma of each sample using the mirVana miRNA isolation kit, according to the manufacturer’s protocol (Applied Biosystems, Foster City, USA).

### Real-Time quantitative RT-PCR of mature miRNAs and data analysis

Individual plasma RNA samples from 56 patients with PTC, 95 with benign thyroid nodules 10 healthy controls, or 7 patients with PTC after tumor resection were polyadenylated by poly (A) polymerase (NEB, Ipswich, MA, USA) and reversely transcribed to cDNA using 50 pmol primer (GCTGTCAACGATACGCTACGTAACGGCATGACAGTGTTTTTTTTTTTTTTTTTTTTTTTV) and the PrimeScript RT reagent kit (Takara, Shiga, Japan), according to the manufacturers’ instruction. The transcribed cDNAs were diluted to 1:5 and used as the templates for further PCR reaction using the SYBR Green PCR Kit (Takara, Shiga, Japan) in Roche 480 Real-Time PCR System (Roche, Basel, Switzerland). The primer sequences were designed based on the miRNA sequences obtained from the miRBase database and the sequences of primers used in qRT-PCR are shown in S1 Table. The PCR amplification was performed in triplicate at 95°C for 5 min and subjected to 40 cycles of 95°C for 5 s, 60°C for 30 s. The RNA diluent was used to replace the template as negative controls and U6 RNA (GenBank accession no. NR 004394) was used as an endogenous control. In addition, 27 PTC and 43 benign nodule tissue samples were ground in liquid nitrogen and their total RNA was extracted using Trizol, according to the manufacturers’ instruction (Invitrogen). After quantification and qualification, the RNA samples were polyadenylated and reversely transcribed into cDNA. U48 RNA (GenBank accession no. NR 004394) was used as an endogenous control for quantitative RT-PCR analysis of the relative levels of miRNA expression in these tissue samples [17]. The relative levels of miRNA expression were calculated by the 2^ΔΔCT^ method. The PCR products were also examined by electrophoresis on 20% PAGE gels.

### Statistical analysis

Data are expressed as the mean ± SD. The difference in the levels of each miRNA between two groups was determined by the Student’s t-test. The diagnostic values of the levels of each miRNA were analyzed by the receiver operating characteristic (ROC) curves. All statistical analyses were performed using SPSS software (version 16.0). A two-sided *P* value of <0.05 was considered statistically significant.

## Results

### Microarray analysis of the miRNA expression profile

**Fig 1:**
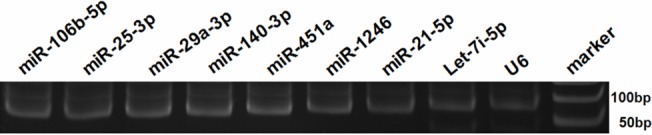
Electrophoresis analysis of miRNAs. The relative levels of plasma each miRNA were determined by qRT-PCR and the PCR products of individual miRNAs were analyzed by PAGE on 20% gels. Data are representative images from three separate experiments.

We used Agilent Human miRNA Microarray to measure the differential expression of plasma miRNAs between patients with PTC and benign thyroid nodules. We detected 166 miRNAs in the plasma samples from PTC patients and 212 from the benign nodule patients (S1 Fig). Further analysis revealed that there were three miRNAs with significantly down-regulated expression and eight with up-regulated expression in the plasma samples from PTC patients (Table 1). The Microarray results indicated that levels of plasma miR-25-3p, miR-451a, miR-140-3p and let-7i were significantly higher in the PTC patients than those with benign thyroid nodules (131.66, 12.66, 25.52, and 3.06 folds, respectively, *P<*0.05). These significantly increased levels of miRNAs were miR-25-3p, miR-29a-3p, miR-21-5p, miR-106b-5p, miR-451a, miR-140-3p, let-7i and miR-1246 and were further validated by qRT-PCR. The PCR products were resolved by PAGE (Fig 1). Furthermore, the levels of plasma miR-320b, miR-4454, miR-5100 were down-regulated, as compared with that in the controls and they were hard to detect in the PTC patients (data not shown).

**Table 1:** Differentially expressed miRNAs in the plasma of patients with PTC or benign thyroid nodules.

*systematic_name*	*p value*	*FC (abs)*	Regulation*
**hsa-miR-1246**	**6.94E-04**	**28.101511**	**up**
**hsa-miR-140-3p**	**0.003152866**	**25.518076**	**up**
**hsa-miR-25-3p**	**4.97E-04**	**131.66217**	**up**
**hsa-miR-29a-3p**	**1.00E-04**	**30.128647**	**up**
hsa-miR-320b	0.023719033	2.2485068	down
hsa-miR-4454	0.046859264	2.0916328	down
**hsa-miR-451a**	**0.013229499**	**12.65919**	**up**
hsa-miR-5100	7.72E-04	3.7727375	down
**hsa-miR-21-5p**	**0.04801573**	**27.59054**	**up**
**hsa-miR-106b-5p**	**0.04214122**	**20.16257**	**up**
**hsa-let-7i-5p**	**0.04235813**	**3.068549**	**up**

*The results indicated that the levels of plasma miR-25-3p, miR-451a, miR-140-3p and let-7i were significantly higher in the PTC patients than those with benign thyroid nodules (131.66, 12.66, 25.52, and 3.06 folds, respectively, *P<*0.05).

### Expression profiles of eight plasma miRNA

**Fig 2:**
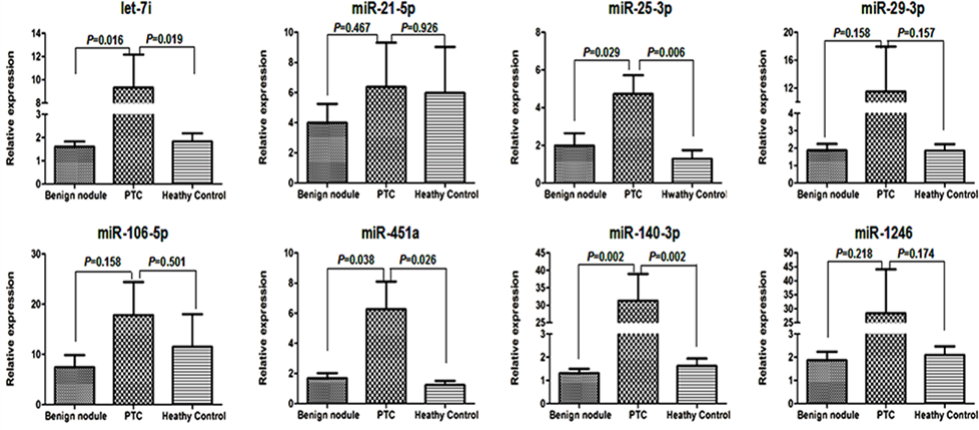
Quantitative analysis of the levels of plasma miRNAs. The relative levels of plasma indicated miRNAs were determined by qRT-PCR indicated. Data are expressed as the means ± SD of each group of samples from three separate experiments. PTC: patients with PTC (n = 56); Benign nodules: patients with benign thyroid nodules (n = 95); Healthy controls (n = 10).

We tested the levels of plasma miR-25-3p, miR-29a-3p, miR-21-5p, miR-106b-5p, miR-451a, miR-140-3p, let-7i and miR-1246 in all 161 plasma samples by qRT-PCR. Primer sequences and characterization of significant miRNAs in qRT-PCR validation were shown in S1 Table and S2 Table. The results indicated that levels of plasma miR-25-3p, miR-451a, miR-140-3p and let-7i were significantly higher in the PTC patients than those with benign thyroid nodules (2.40, 3.69, 23.83, and 5.72 folds, respectively, *P<*0.05) and the healthy controls (3.67, 5.08, 19.27 and 5.10 folds, respectively, *P<*0.05, Fig 2). However, there were no significant difference in the levels of plasma miR-29a-3p, miR-21-5p, miR-106b-5p and miR-1246 between the PTC patients and those with benign nodules or the healthy controls.

### Expression levels of four miRNAs in the tissue samples

**Fig 3:**
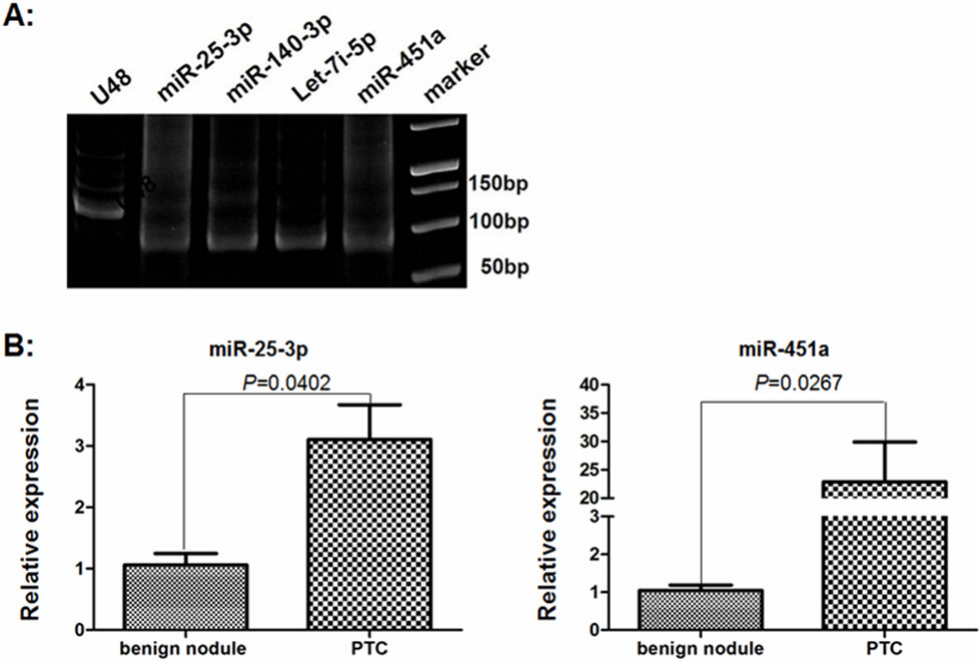
The relative levels of miRNA expression in the thyroid tissues. The relative levels of miR-25-3p, miR-140-3p, Let-7i-5p and miR-451a expression in 27 PTC and 43 benign thyroid nodule tissues were determined by qRT-PCR and the PCR products were analyzed by PAGE on 20% gels. Data are representative images and expressed as the means ± SD of each group of samples from three separate experiments. A: Electrophoresis analysis of PCR products. B: Quantitative analysis of the levels of miR-25-3p and miR-451a expression in the PTC and benign thyroid nodules. There was no significant difference in the relative levels of miR-140-3p and Let-7i-5p expression in the thyroid tissue samples between these two groups of patients (data not shown).

Giving that the levels of plasma miR-25-3p, miR-451a, miR-140-3p and let-7i were significantly higher in the PTC patients than in those with benign nodules and the healthy subjects, we further examined their expression in the PTC and benign nodule tissue samples by qRT-PCR (Fig 3A). The levels of miR-451a and miR-25-3p in the PTC tissues were 22.8-fold (P = 0.0267) and 3.1-fold (P = 0.0402) higher than that in the benign nodule tissues (Fig 3B). However, there was no significant difference in the levels of miR-140-3p and let-7i expression between the PTC and benign nodule tissues from this population (data not shown).

### Predictive value of the levels of plasma miRNA

**Fig 4:**
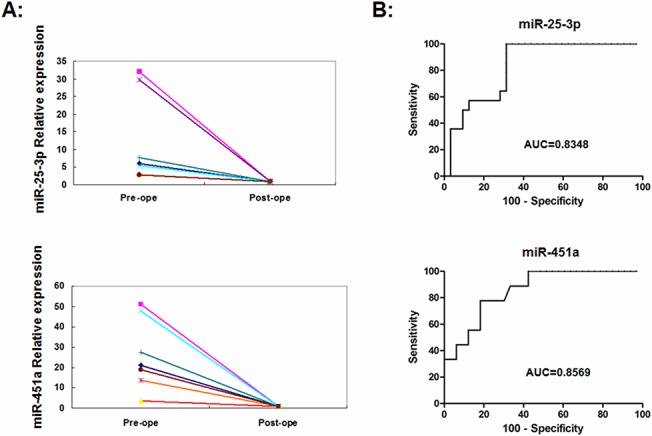
The diagnostic values of plasma miR-25-3p and miR-451a. The levels of plasma miR-25-3p and miR-451a in seven patients were determined before (pre-ope) and after surgical removal of the tumor (post-ope). **A:** The changes in the levels of plasma miR-25-3p and miR-451a in PTC patients. Data are the mean values of individual patients before and after surgery. The relative levels of plasma miR-25-3p or miR-451a before surgery were calculated by 2^-ΔΔct^ method and their relative levels after surgery were designated as 1. **B:** ROC curves. The diagnostic values of the levels of plasma miR-25-3p and miR-451a were determined by ROC analysis. AUC: Area under the curve.

Considering the expression of miR-25-3p and miR-451a in plasma as potential biomarkers for PTC, we measured the plasma levels of these miRNAs in seven PTC patients before and 4–7 d after tumor excision by qRT-PCR. The levels of plasma miR-25-3p and miR-451a significantly decreased after surgery in all seven patients, as compared with that before surgery (P = 0.028, P = 0.004, respectively, Fig 4A). To evaluate the diagnostic values of plasma miR-25-3p and miR-451a for PTC, ROC curve analysis was performed in 56 patients with primary PTC and 95 patients with benign thyroid nodules (Fig 4B). A comparison of the PTC with those with benign nodules indicated that the levels of plasma miR-25-3p had an AUC of 0.835 [95% confidence interval (CI) = 0.720–0.950] and miR-451a an AUC of 0.857 [95% CI = 0.728–0.963]. At the cut-off values of 1.41 for miR-25-3p, and 1.38 for miR-451a, their sensitivity and specificity were 92.8% and 68.8%, 88.9% and 66.7%, respectively. Both miRNAs at the cut-off value of 1.25 had an AUC of 0.863 [95% CI = 0.788–0.938] with a sensitivity of 95.6% and specificity of 64.1% respectively. Combining these two miRNAs did not result in an improved AUC and higher sensitivity or specificity. Hence, the levels of plasma miR-25-3p and miR-451a may be valuable in distinguishing patients with PTC from those with benign nodules.

## Discussion

The FNAC is the common method for preoperatively differential diagnosis of thyroid benign nodules from malignant thyroid nodules; however, the predictive value of FNAC is still limited in subjects with cytological features of suspicious malignancy [18]. Previous studies have reported that circulating miRNAs are potential biomarkers for the diagnosis of numerous cancers [19–23]. While previous studies examined differentially expressing miRNAs mainly in tumor tissues from PTC patients [11–13] there are a few reports on the possible utility of circulating miRNA quantification in patients with PTC [14–16]. In the present study, we first screened the varying levels of plasma miRNAs in patients with PTC or benign nodules by the miRNA Microarray. We found significantly higher levels of eight plasma miRNAs and lower levels of three plasma miRNAs in the PTC patients, related to that in the patients with benign nodules. We further validated significantly higher levels of plasma miR-25-3p, miR-451a, miR-140-3p and let-7i in the PTC patients, as compared with that in the patients with benign nodules or the healthy controls by qRT-PCR. Furthermore, we found that the miR-451a and miR-25-3p, but miR-140-3p and let-7i expression, were up-regulated in the PTC tissues. The high levels of circulating miR-451a and miR-25-3p we identified in the PTC patients were differ from those found in the PTC patients in Southern China [14] as well as in Caucasians [15,16]. These findings support the notion that varying miRNAs are sensitive biomarkers for the pre-operative diagnosis of PTC in different regions of PTC patients. More importantly, ROC analyses revealed that the levels of plasma miR-25-3p at cut-off 1.41 and miR-451a at 1.38 had a sensitivity and reasonable specificity for distinguishing PTC from benign nodules, respectively. Therefore, the levels of plasma miR-451a and miR-25-3p may serve as new minimally invasive biomarkers for the preoperative diagnosis of PTC in Northern China.

The roles of miR-25-3p and miR-451a in tumorigenesis have been explored in many studies, but their oncogene roles remain controversial. Higher levels of miR-451 expression are associated with the tumor recurrence and poor survival of patients with gastric cancer [20]. Furthermore, higher levels of circulating miR-451 are detected in patients with gastric cancer and their levels are significantly reduced in those patients after tumor removal [22]. In contrast, significantly lower levels of serum miR-451 are detected in breast cancer patients and miR-451 acts as one of the blood-based biomarkers for the diagnosis of breast cancer [23]. Furthermore, miR-451 has been reported to inhibit the growth and invasion of glioma cells by down-regulating the PI3K/AKT activation [21]. The miR-25 may have an oncogenic activity because higher levels of miR-25 have been detected in adjacent non-tumor of epithelial ovarian cancer tissue, female lung adenocarcinoma, gastric cancer tissues and cell lines, and malignant cholangiocarcinoma [24–28]. The levels of miR-25 expression are positively correlated with tumor stages, histologic degrees, and regional lymph node involvement in some kind of cancers [24–28]. Furthermore, significantly higher levels of serum miR-25 are detected in patients with varying types of malignancies, including breast cancer, esophageal squamous cell carcinoma and HBV-positive hepatocarcinoma and the levels of serum miR-25 is a sensitive biomarker for minimally invasive diagnosis of these tumors [29–31]. In contrast, significantly lower levels of miR-25 are detected in human colon cancer tissues when compared to those in matched, non-neoplastic mucosa tissues and restoration of miR-25 expression inhibits the proliferation and migration of colon cancer cells, suggesting that miR-25 may function as a tumor suppressor in colon cancer [32]. Therefore, miR-451 and miR-25 may have different regulatory roles during the development of different tissues of tumors.

Previous studies have shown that upregulated miR-451 expression in the thyroid tissue is associated with PTC lymphatic metastasis [33] while ectopic expression of miR-25 in anaplastic thyroid carcinoma cells inhibits the proliferation of cancer cells by inducing cell-cycle arrest at G2/M-phase [34]. In our present study, we detected significantly higher levels of miR-451a and miR-25-3p in patients with PTC, as compared to that in the patients with benign thyroid nodules or in the healthy controls and the higher levels of plasma miR-25-3p and miR-451a decreased after surgical removal of the tumor in PTC patients. Furthermore, we detected significantly higher levels of miR-451a and miR-25-3p expression in the PTC tissues than that in the benign nodules. Our data were in disagreement with the previous findings [34] and the discrepancy among different studies on the role of miR-25 in thyroid cancer may stem from the varying roles of miR-25 in regulating the development of different histological types of thyroid cancers. It is well known that circulating miRNAs are products of tumor cell death and lysis, released by tumor-derived microvesicles or exosomes and may come from cancer-related immune responses [14,35,36]. The higher levels of plasma miR-25-3p and miR-451a may be released by PTC.

We recognized that our study had limitations, including a small sample size, without detail analysis of the potential relationship between the levels of plasma miR-25-3p or miR-451a and the clinicopathological features due to small sample size for difficult stratification and the lack of functional study of these miRNAs. Particularly, the sample size for the miRNA array was small so that the array results were not conclusive and only provided a clue for the subsequent analyses. Thus, further studies in a new bigger population are needed to evaluate the values of the plasma miR-25-3p and miR-451a levels as biomarkers for the preoperative diagnosis of PTC as well as for predicting the tumor recurrence.

## Conclusions

In conclusion, our data indicated that the levels of circulating miR-25-3p and miR-451a were significantly higher in the PTC patients than that in those with benign thyroid nodules or healthy controls and they were highly expressed in the PTC tissues. The levels of plasma miR-25-3p and miR-451a were reduced after surgical removal of the tumor in PTC patients. Further analysis revealed that the levels of plasma miR-25-3p at cut-off 1.41 and miR-451a at 1.38 had a sensitivity and a reasonable specificity for distinguishing PTC from benign nodules, respectively. Therefore, our findings may provide a new basis for the development of an easy, minimally invasive diagnostic tool for the preoperative differentiation of thyroid nodules.

## Supporting Information

S1 Fig Microarray assay of varying levels of plasma miRNAs in patients with PTC and those with benign thyroid nodules. Six plasma samples from three PTC patients and three with benign thyroid nodules selectively randomly. Their RNAs were extracted and subjected in triplicate to microarray analysis. The intensity of hybridization signals was converted and the unsupervised hierarchical clustering of 2008 miRNAs (rows) in individual samples are shown in individual columns. The z-score across the top bar illustrates the relative expression level of a miRNA: green for low expression and red for high expression. Data ae expressed as the mean values of individual samples.
(TIF)

S1 Table Primer sequences and amplicon length used in qRT-PCR. (DOC)

S2 Table Characterization of significant miRNAs in qRT-PCR validation. (DOC)
