# Correction: Circulating miR-25-3p and miR-451a May Be Potential Biomarkers for the Diagnosis of Papillary Thyroid Carcinoma

**DOI:** 10.1371/journal.pone.0135549

**Published:** 2015-08-12

**Authors:** Min Li, Qingbin Song, Hang Li, Yi Lou, Lili Wang

The second author’s name is spelled incorrectly. The correct name is: Qingbin Song. The citation is correct.
