# Supplementary figures and images for: Circulating miR-25-3p and miR-451a May Be Potential Biomarkers for the Diagnosis of Papillary Thyroid Carcinoma
Source: PLoS One. 2015 Jul 13;10(7):e0132403. doi: 10.1371/journal.pone.0132403 (PMC4500410; doi:10.1371/journal.pone.0132403)

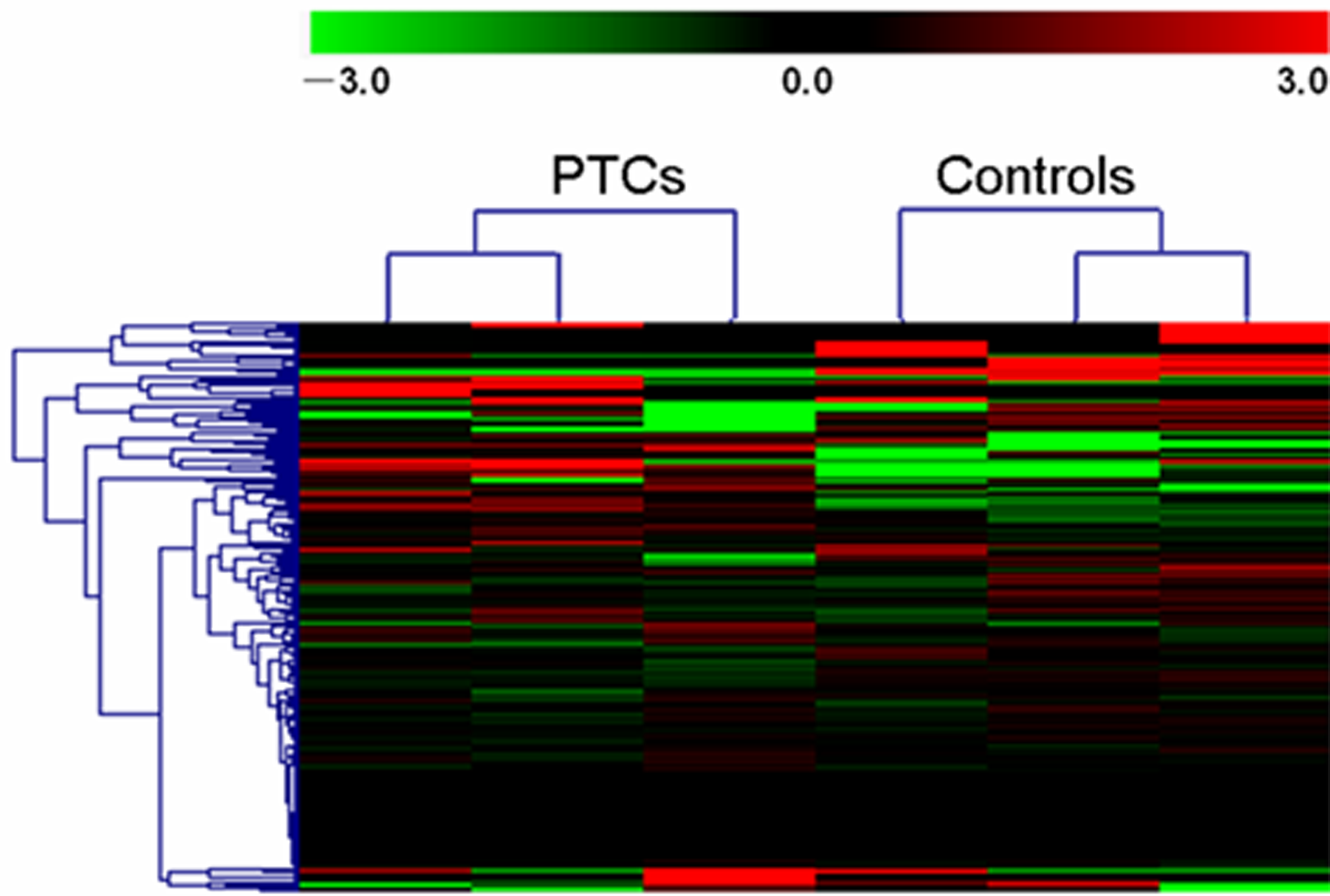

Supplement: S1 Fig — Six plasma samples from three PTC patients and three with benign thyroid nodules selectively randomly. Their RNAs were extracted and subjected in triplicate to microarray analysis. The intensity of hybridization signals was converted and the unsupervised hierarchical clustering of 2008 miRNAs (rows) in individual samples are shown in individual columns. The z-score across the top bar illustrates the relative expression level of a miRNA: green for low expression and red for high expression. Data ae expressed as the mean values of individual samples. (TIF) [file pone.0132403.s001.tif]
