# Supplementary material for: Circulating miR-25-3p and miR-451a May Be Potential Biomarkers for the Diagnosis of Papillary Thyroid Carcinoma
Source: PLoS One. 2015 Jul 13;10(7):e0132403. doi: 10.1371/journal.pone.0132403 (PMC4500410; doi:10.1371/journal.pone.0132403)
Supplement: S1 Table — (DOC) [file pone.0132403.s002.doc]

**S1 Table Primer sequences and amplicon length used in qRT-PCR.**

| **miRNA** | **Primer seqTences (5’-3’)** | **Amplicon length (bp)** |
| --- | --- | --- |
| **miR-1246** | AATGGATTTTTGGAGCAGG | 79bp |
| **miR-140-3p** | TACCACAGGGTAGAACCACG | 80bp |
| **miR-25-3p** | ATTGCACTTGTCTCGGTCTG | 80bp |
| **miR-29a-3p** | TAGCACCATCTGAAATCGG | 79bp |
| **miR-21-5p** | TAGCTTATCAGACTGATGTTGA | 82bp |
| **let-7i-5p** | TGAGGTAGTAGTTTGTGCTGTT | 82bp |
| **miR-106b-5p** | TAAAGTGCTGACAGTGCAGAT | 81bp |
| **miR-451a** | AAACCGTTACCATTACTGAG | 81bp |
| **RNU48** | AGTGATGATG ACCCCAGGTA A | 122bp |
| **Antisense:** | GCTGTCAACGATACGCTACG |  |
