# Supplementary material for: Circulating miR-25-3p and miR-451a May Be Potential Biomarkers for the Diagnosis of Papillary Thyroid Carcinoma
Source: PLoS One. 2015 Jul 13;10(7):e0132403. doi: 10.1371/journal.pone.0132403 (PMC4500410; doi:10.1371/journal.pone.0132403)
Supplement: S2 Table — (DOC) [file pone.0132403.s003.doc]

**S2 Table Characterization of significant miRNAs in qRT-PCR validation.**

| **systematic_name** | **Accession no.** | **Chr** | **miRNA sequence** |
| --- | --- | --- | --- |
| **hsa-miR-140-3p** | MIMAT0004597 | chr16 | uaccacaggguagaaccacgg |
| **hsa-miR-25-3p** | MIMAT0000081 | Chr7 | cauugcacuugucucggucuga |
| **hsa-miR-451a** | MI0001729 | chr17 | aaaccguuaccauuacugaguu |
| **hsa-let-7i-5p** | MIMAT0000415 | chr12 | ugagguaguaguuugugcuguu |
